# The Effect of Spironolactone on β-amyloid-Induced Memory Impairment in Male Rats: The Role of Microglial Inhibition

**DOI:** 10.34172/apb.2022.065

**Published:** 2021-09-29

**Authors:** Mohammad Mehdipour, Masoumeh Emamghoreishi, Majid Reza Farrokhi, Elahe Amirinezhadfard, Mojtaba Keshavarz

**Affiliations:** ^1^Department of Neuroscience, School of Advanced Medical Sciences and Technologies, Shiraz University of Medical Sciences, Shiraz, Iran.; ^2^Department of Pharmacology, School of Medicine, Shiraz University of Medical Sciences, Shiraz, Iran.; ^3^Shiraz Neuroscience Research Center, Shiraz University of Medical Sciences, Shiraz, Iran.

**Keywords:** Beta-amyloid, Iba1, Microglial activation, Memory, Neuroinflammation, Spironolactone

## Abstract

***Purpose:*** Neuroinflammation was indicated in the pathophysiology of Alzheimer’s disease (AD). Previous reports have also signified that spironolactone has anti-inflammatory effects. Therefore, the aim of this study was to assess the modulatory effects of spironolactone on neuroinflammation and memory loss in a rat model of AD.

***Methods:*** The β-amyloid protein fragment 25-35 (Aβ) was injected in the dorsal hippocampus (5 μg/2.5 μL each side) of male Sprague-Dawley rats for four consecutive days to induce memory impairment. Animals have intraperitoneally received spironolactone (10, 25, or 50 mg/kg, N = 6/ group) or vehicle for 14 days. The passive inhibitory avoidance and the novel recognition tests were used for memory evaluation. Neuroinflammation was assessed by measuring the level of Iba1 protein, a marker of microglial activation, using western immunoblotting.

***Results:*** Different doses of spironolactone showed no significant changes in latency times and discriminations ratios in passive inhibitory avoidance and novel recognition tests, respectively, as compared to vehicle. However, spironolactone-treated groups showed significantly lower Iba1 protein levels in comparison to the vehicle-treated group (*P* < 0.01).

***Conclusion:*** Spironolactone had a modulatory effect on neuroinflammation through a repressive effect on microglial activation with no valuable effect on memory improvement in a rat model of AD. The findings of this study suggest that Aβ-induced memory loss may not be directly linked to microglial activation. Spironolactone may be a potential candidate to be examined in other neuroinflammatory disorders.

## Introduction

Alzheimer’s disease (AD) is a widespread neurodegenerative disorder around the world. AD patients suffer from cognitive declines and disturbances in several aspects of neuropsychiatric function.^1^ Despite rigorous research, the exact pathophysiology of AD is still unknown.^1^ As such, AD treatment is currently a serious challenge in medicine. Understanding the pathophysiology of AD is a helpful way to develop new treatments for AD. However, targeting the β-amyloid (Aβ), the known contributor of neurodegeneration in AD,^2^ has not led to new treatments to arrest disease progression.^3^ Therefore, recent researches have been focused on neuroinflammation, a dominant characteristic of AD, manifested by hyperactivity of astrocytes and microglia.^4^

Microglia are considered to have immune functions in the central nervous system (CNS).^5^ Pathological changes in the brain activate microglia and provoke inflammatory responses in the CNS.^6^ The reinforced inflammatory reactions have been considered as a risk factor for neurodegenerative disorders.^6^ It has been suggested that Aβ aggregation causes severe neuroinflammation in the affected brain areas in AD.^7^ Thus, chronic microglial activation by Aβ increases the chance of Aβ deposition, synapse loss,^8^ pro-inflammatory and neurotoxic substances, and Aβ spreading throughout the CNS.^9,10^ The interaction of microglia and Aβ also produces several other deleterious effects.^5^ These pathological conditions accelerate neurodegenerative processes and exaggerate neuronal loss.^5^ Therefore, microglia may be a possible target in the treatment of neurodegeneration like AD.

There are various anti-inflammatory agents. Epidemiological studies propose that use of anti-inflammatory drugs decrease the incidence of AD. However, clinical trials based on nonsteroidal anti-inflammatory drugs in patients suffered from AD were unsatisfactory.^11^ Also corticosteroids have multiple side effects and we could not administer corticosteroids for chronic diseases like AD. Spironolactone, an aldosterone mineralocorticoid receptor antagonist, generally is a good choice in clinic for reducing mortality and morbidity of patients in chronic disorders like cardiovascular diseases.^12^ It has been shown that spironolactone produced anti-inflammatory effects in the peripheral tissues and CNS.^13^ Hence, human studies have also shown that spironolactone suppressed inflammatory mediators like tumor necrosis factor-α (TNF-α) and monocyte chemoattractant protein-1 and decreased the transcription of inflammatory genes in monocytes.^14^ Recent evidence has indicated that the neuromodulatory effects of spironolactone may positively influence neurological disorders. Thus, spironolactone has decreased the infarct size in an animal model of stroke.^15^ Also, spironolactone has been shown to positively affect memory in obese patients.^16^ Moreover, our previous study demonstrated that spironolactone protected neuronal and glial cells against N-methyl-D-aspartate toxicity in cell culture.^17^ Furthermore, spironolactone inhibited microglial activation and decreased pro-inflammatory markers in an animal model of radicular pain.^18^ These evidences suggest that spironolactone with its anti-inflammatory effects may have beneficial effects on neurological disorders.

Taken together, considering the inhibitory effect of spironolactone on microglia activation and the contribution of the hyperactivation of microglia in the pathophysiology of AD, we proposed that spironolactone may improve cognitive functions in AD by suppressing microglial activation. Therefore, this study was intended to evaluate spironolactone effects on microglial activation and memory in an Aβ animal model of AD using ionized calcium-binding adaptor molecule 1 (Iba1) as a selective indicator of microglial-associated neuroinflammation.^19-21^

## Materials and Methods

### Materials

The materials used in this study included: Aβ protein fragment 25-35 and spironolactone powder (Sigma-Aldrich, USA); anti-mouse Iba1 monoclonal antibody and PVDF membrane (Santa Cruz Biotechnology Company, USA); anti-rabbit β-actin monoclonal antibody and anti-mouse IgG horseradish peroxidase (HRP)-linked and anti-rabbit IgG HRP-linked secondary antibodies (Cell Signaling Technology Company, USA); Enhanced chemiluminescence (ECL) kit (GE Healthcare Life Sciences, UK); bicinchoninic acid assay (BCA) kit (DNA Biotechnology Company, Iran); and pre-stained protein ladder (Thermo Scientific, UK).

Freshly prepared spironolactone solution in tween-20 (5% in distilled water as vehicle) was used. Dissolved Aβ (2 μg/μL) in sterile phosphate-buffered saline (PBS) was deposited at minus 70^o^C; and kept at 37°C for 4 days to form fibril aggregation before use.

### Animals

Male Sprague-Dawley rats (200-250 g; N = 42) were bought from Shiraz University of Medical Sciences. Rats were accommodated in cages with woodchip bedding (two/Plexiglas cage) at 20-22˚C temperature and 12 hours:12 hours light/dark cycles and could freely approach standard food and water. All the experiments were performed according to the NIH Guideline for the care and use of laboratory animals with the minimum suffering and numbers of animals.

### Treatment groups

Rats were randomly divided into seven groups (N = 6/group). The groups were as follows: 1) No intervention (NI), 2) PBS plus vehicle (sham), 3) Aβ plus vehicle (Aβ), 4) Aβ plus spironolactone 10 mg/kg (Aβ+s10), 5) Aβ plus spironolactone 25 mg/kg (Aβ+s25), 6) Aβ plus spironolactone 50 mg/kg (Aβ+s50), 7) PBS plus spironolactone 25 mg/kg (sham+s25).

**Figure 1:**
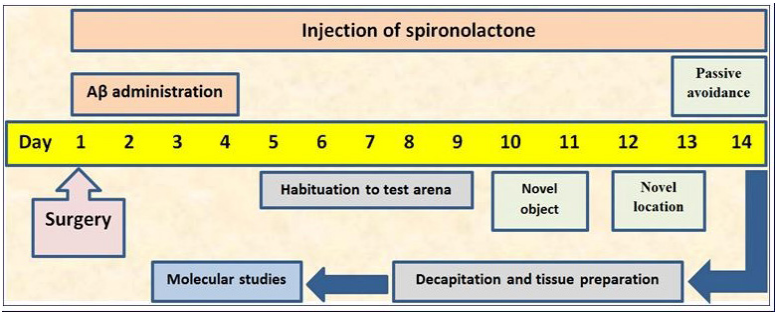
Depiction of study design.

Following the surgery (described below), rats were intraperitoneally (i.p.) administered spironolactone (groups 4-7) or vehicle (groups 2 and 3) for 14 days (Figure 1). The spironolactone doses were chosen based on previous reports.^22,23^

### Surgery and cannulation for β-amyloid injection

The anesthetized rat (ketamine 100/xylazine10 mg/kg mixture, i.p.) was bilaterally inserted a stainless steel guide cannula (22 gauges) in CA1 region of the dorsal hippocampus (AP -3.8, ML ± 2.2, DV – 2.7). The cannula was attached to the skull by stainless screws and acrylic cement. Then, the aggregated Aβ peptide was bilaterally injected (5 μg/2.5 μL/5 min each side) into the CA1 region of the dorsal hippocampus for 4 consecutive days to induce memory impairment.^24^ Sham and sham+s25 groups had also the surgery but received PBS.

### Passive inhibitory avoidance test

The memory was evaluated by a passive inhibitory avoidance test as described by Venault et al.^25^ with some adjustments. Briefly, the shuttle box consists of two equal-sized compartments (20×20×30 cm; illuminated and dark) detached by a guillotine door. After habituation for 15 seconds in the illuminated chamber, rats were allowed to enter the dark chamber and the door was shut. Animals that did not move into the dark chamber after 120 seconds were eliminated from the study. Thirty minutes after the habituation trial, the rat was positioned in the illuminated chamber and when the rat arrived in the dark chamber the door was closed and a foot shock was delivered (Intensity = 1.5 mA, Frequency = 50 Hz, Duration = 3 seconds) through rods of stainless steel grids (acquisition trial). The rat was returned to its home cage after 15 seconds and tested again two minutes later. Rats that remained in the illuminated chamber for more than 120 seconds were considered as successful acquisition of avoidance. Otherwise, the rat was excluded from the study. The retention trial was executed twenty-four hours later that was similar to the acquisition trial but with no delivery of foot shock. The time delay to move into the dark chamber in the absence of electric shock was recorded (step-through latency time) as an indicator of inhibitory avoidance memory. Three hundred seconds was the cut-off time for moving into the dark chamber during the retention trial.

### Novel recognition tests

The field for exploration (40×80×100 cm) was made of wood and its bed was covered with sawdust. Animal behaviors were monitored and recorded by a camera and video recorder for later analysis. Objects with different shapes, colors and sizes (9×8×7 cm to 25×15×10 cm) and too weighty for the animals to displace were presented as stimuli.^26^

### Novel object preference test (what memory)

Before the onset of the behavioral test, animals were accustomed to the field with no object for 5 days (10 min/d).^26^ The test included an acquisition phase and a recognition test executed with a time interval of 90 minutes or 24 hours for assessing the short- or long-term memory, respectively. In the acquisition phase, two similar objects (for example, A and A′) were located 10 cm from the opposite walls in the field. The rat was placed in the field facing the adverse wall and allowed 30 seconds to explore A and A′ or 5 minutes in the arena. When the rat’s nose was toward the object at a distance of < 2 cm, it was considered as an exploration behavior. Other activities such as sitting on or resting against the objects or looking around were not taken into account as exploration behaviors. Ninety minutes and 24 hours after the acquisition phase, the recognition phase was performed. During the 3-minute recognition test, the animal was positioned in the field and displayed with two objects (one being the same as used in the acquisition phase, e.g. A, and the other one being novel) located at the same locations as at the acquisition phase. The novel objects used for the short-term memory and long-term memory tests were different (e.g. B and C, respectively) (Figure 2). The discrimination ratio was determined as follow:

#### Time near the new object – time near the familiar object / time near the new object + time near the familiar object.

**Figure 2:**
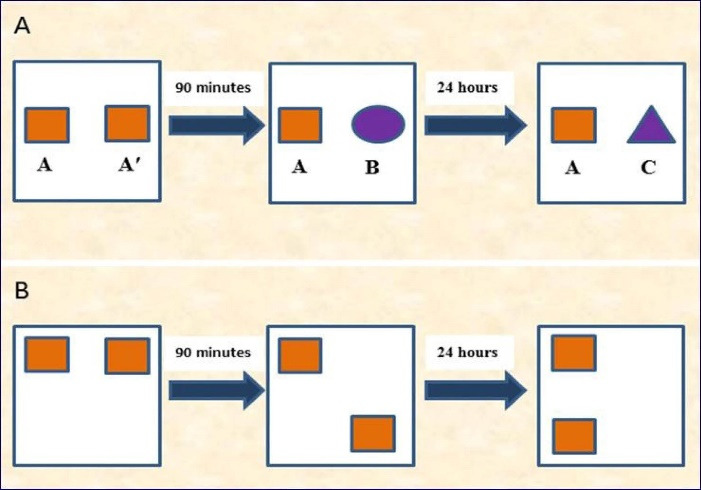
(A): Illustration of the novel object preference test (what memory), and (B): The novel location test (where or spatial memory).

### Novel object location test (where memory)

This test was used to assess the rat’s spatial memory and ability to recognize novel location. In the 5-minute acquisition phase, the animal was allowed to explore two objects (A and A′) placed on the opposite sides of the arena. The animal’s exploration time for each object was recorded. The test phase was performed after a delay of 90 minutes or 24 hours for the assessment of short- and long-term memory, respectively. In the test phase, both objects were equally familiar but one was in a new location (Figure 2).^26^ The discrimination ratio was estimated as follow:

#### Time near the object in new location – time near the object in previous location / Time near the in new location + time near the object in previous location.

### Tissue preparation

After the completion of behavioral tests, animals were beheaded under anesthetization by chloroform. The hippocampi of animals were then quickly isolated on ice and stored at -80°C up to use for western immunoblotting.^24^

### Total protein determination

The hippocampi were homogenized on ice using cold NP-40 lysis buffer containing protease and phosphatase inhibitor cocktail. The lysates were centrifuged at 12000 g for 20 minutes at 4°C to remove debris. Total protein concentrations were estimated by the BCA assay kit in accordance with the manufacturer’s instruction.^27^

### Determination of Iba1 protein levels

Western immunoblotting was used to determine the level of Iba1 protein as a marker of microglial activation. From each sample, 30μg total protein was loaded into the 5%-12% stacking resolving SDS polyacrylamide gel (SDS-PAGE). The gel was run at 110V for 1 hour; and the separated proteins were transferred onto the PVDF membrane using the Semi-dry transblotting method at 15V for 30 minutes. The blot was then blocked in 5% BSA solution for one hour followed by overnight incubation at 4°C with Iba1 primary antibody (1:200 dilution in phosphate-buffered saline (PBST) with 0.1% Bovine serum albumin (BSA)). The following day, the blot was washed with PBST solution and incubated for 1 hour at room temperature with anti-mouse HRP-conjugated secondary antibody (1:5000 dilution in PBST with 0.1% BSA). For immunodetection of β-actin, an internal control, the blot was blocked in 5% BSA solution for one hour followed by overnight incubation at 4°C in beta-actin antibody (1:8000 dilution in PBST with 0.1% BSA). The following day, the blot was washed with PBST solution and incubated for 1 hour with anti-rabbit HRP-conjugated secondary antibody (1:12000 dilution in PBST with 0.1% BSA) at room temperature. Following washing with PBST, the blots were visualized by an ECL detection kit and Bio-Rad Chemi-Doc MP imaging system using Image Lab software. Then the images were analyzed by ImageJ software.^24,27^

### Statistical analysis

Data are presented as the mean + SEM (standard error of mean). Normal Distribution of data was evaluated by the Kolmogorov-Smirnov test. The data were analyzed using one-way analysis of variance (ANOVA) followed by the LSD (for equal variance) or Tamhane’s T2 (for unequal variance) as post hoc tests. SPSS software version 23 was used for data analysis and a *P* < 0.05 was considered as a significance level.

## Results and Discussion

### Passive inhibitory avoidance test

**Figure 3:**
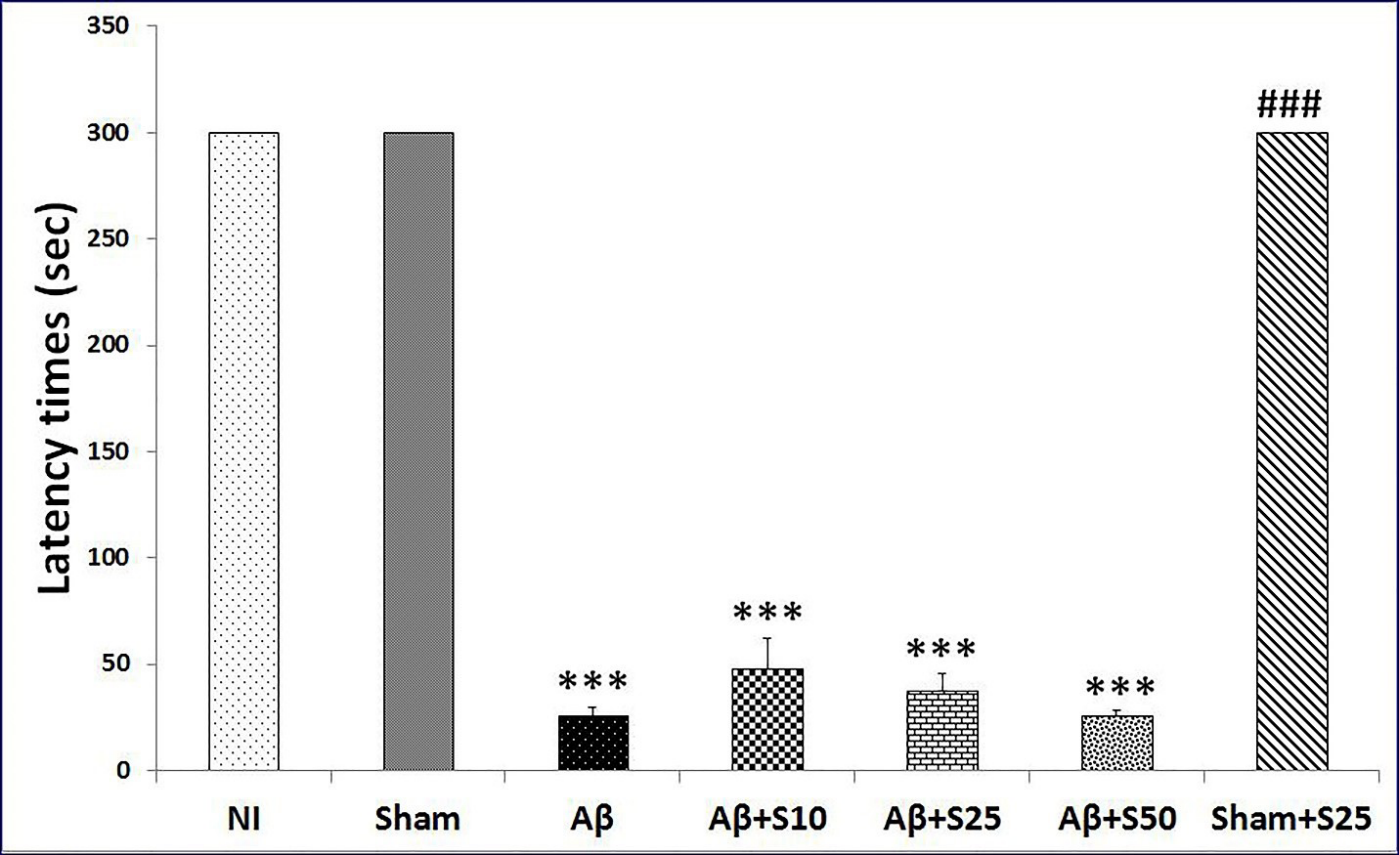
Bar graphs represent the mean + SEM of latency times for no intervention (NI), PBS plus vehicle (sham), Aβ plus vehicle (Aβ), Aβ plus spironolactone 10 mg/kg (Aβ+s10), Aβ plus spironolactone 25 mg/kg (Aβ+s25), Aβ plus spironolactone 50 mg/kg (Aβ+s50) and PBS plus spironolactone 25 mg/kg (sham+s25) (n = 6/group). There were no significant differences in latency times between NI and the sham group and sham+s25 group. However, the Aβ (*P* < 0.001), Aβ+s10 (*P* < 0.001), Aβ+s25 (*P* < 0.001) and Aβ+s50 (*P* < 0.001) groups had significantly lower latency times as compared to the sham group. No significant differences were found in latency times between groups received Aβ + spirolanctone (at all studied doses) in comaprison to Aβ group (control). The latency time was significanty lower in Aβ+s25 group as compared to sham+s25 group (*P* < 0.001). *** P < 0.001 significantly different from NI and sham groups; ### *P* < 0.001 significantly different from Aβ+s25.

Our study detected no significant change in latency times between NI and sham+s25 groups and the sham group. However, the Aβ (*P* < 0.001), Aβ+s10 (*P* < 0.001), Aβ+s25 (*P* < 0.001) and Aβ+s50 (*P* < 0.001) groups had significantly lower latency times as compared to the sham group. No significant distinction were found in latency times between groups received Aβ + spirolancton (at all studied doses) in comaprison to Aβ group (control). The latency time was significanty lower in Aβ+s25 group in comparison to sham+s25 group (*P* < 0.001) (Figure 3). Our results revealed that the intra-hippocampal injection of Aβ impaired learning and memory in rats which is in harmony with the previous reports of memory impairment induced by Aβ in rats.^24,28-31^

### Novel recognition tests

**Figure 4:**
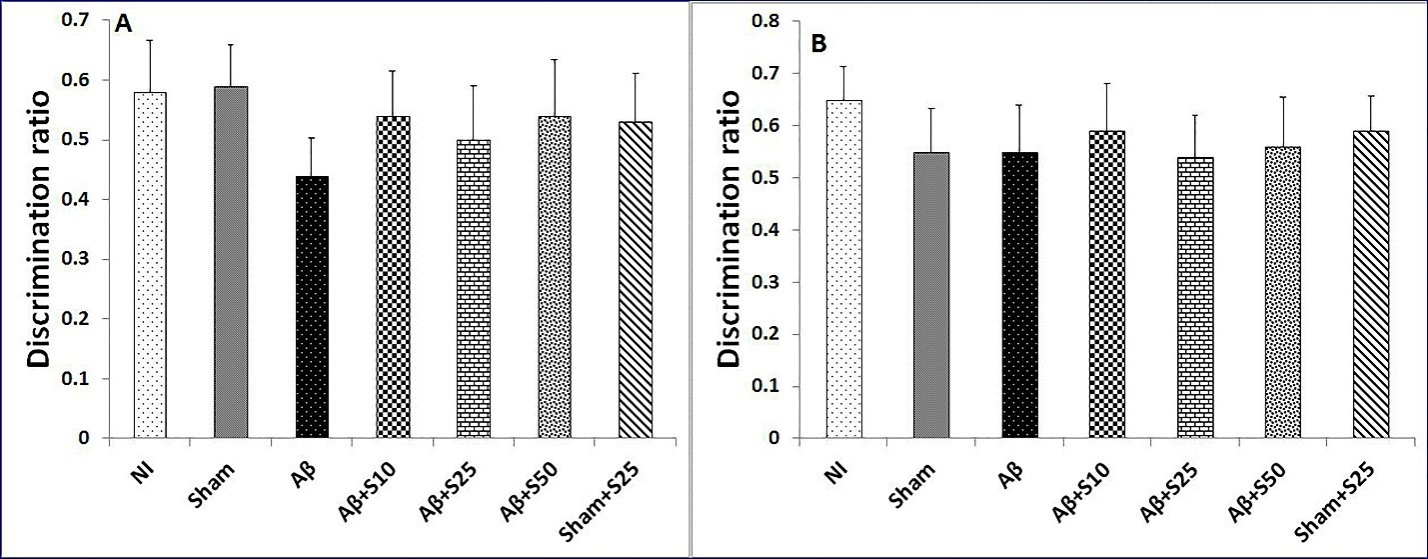
Bar graphs represent the mean + SEM of discrimination ratios for no intervention (NI), PBS plus vehicle (sham), Aβ plus vehicle (Aβ), Aβ plus spironolactone 10 mg/kg (Aβ+s10), Aβ plus spironolactone 25 mg/kg (Aβ+s25), Aβ plus spironolactone 50 mg/kg (Aβ+s50) and PBS plus spironolactone 25 mg/kg (sham+s25) (n = 6/group). No significant differences in discrimination ratios for short term memory (A) and long term memory (B) were found between studied groups in novel object preference test.

No discernable change in discrimination ratios were found between studied groups in short term and long term memory in novel object preference tests (Figure 4).

**Figure 5:**
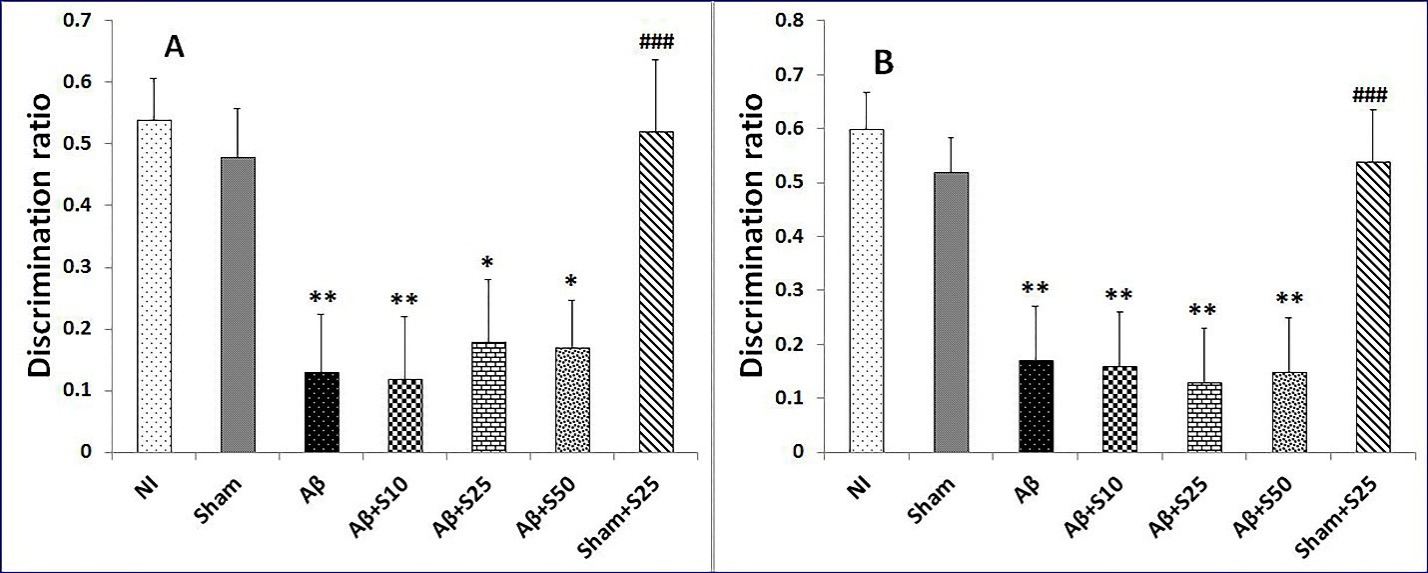
Bar graphs represent the mean + SEM of discrimination ratios for short term memory and long term memory in novel location test for no intervention (NI), PBS plus vehicle (sham), Aβ plus vehicle (Aβ), Aβ plus spironolactone 10 mg/kg (Aβ+s10), Aβ plus spironolactone 25 mg/kg (Aβ+s25), Aβ plus spironolactone 50 mg/kg (Aβ+s50) and PBS plus spironolactone 25 mg/kg (sham+s25) (n = 6/group). (A): In short term memory in novel location test, the discrimination ratios of sham+s25 and NI groups were not significantly different from that of the sham group. However, the Aβ group (*P* < 0.01), Aβ+s10 (*P* < 0.01), Aβ+s25 (*P* < 0.05), and Aβ+s50 (*P* < 0.05) had significantly lower discrimination ratios as compared to the sham group. In addition, Aβ+s25 group showed significantly lower discrimination ratio as compared to sham+s25 group (*P* < 0.001). Nevertheless, there were no significant differences in discrimination ratios between groups received Aβ.(B): In long term memory in novel location test, there were no significant differences in discrimination ratios between the NI, sham and sham+s25 groups. The Aβ (*P* < 0.01), Aβ+s10 (*P* < 0.01), Aβ+s25 (*P* < 0.01), and Aβ+s50 (*P* < 0.01) had significantly lower discrimination ratios as compared to the sham group. There were no significant differences in discrimination ratios between group received Aβ. There was significantly lower discrimination ratio in Aβ+s25 group as compared to sham+s25 group (*P* < 0.001). * *P* < 0.05, ** P < 0.01 significantly different from NI and sham groups; ### *P* < 0.001 significantly different from Aβ+s25.

In short term memory in novel location test, the discrimination ratios of sham+s25 and NI groups showed no significant change copmared to the sham group. However, the Aβ group (*P* < 0.01), Aβ+s10 (*P* < 0.01), Aβ+s25 (*P* < 0.05), and Aβ+s50 (*P* < 0.05) had significantly lower discrimination ratios as compared to the sham group (Figure 5). In addition, Aβ+s25group showed significantly lower discrimination ratio as compared to sham+s25 group (*P* < 0.001) (Figure 5). Nevertheless, there were no significant differences in discrimination ratios between groups received Aβ.

In long term memory in novel location test, there were no discernable change in discrimination ratios between the NI, sham and sham+s25 groups. The Aβ (*P* < 0.01), Aβ+s10 (*P* < 0.01), Aβ+s25 (*P* < 0.01), and Aβ+s50 (*P* < 0.01) had significantly lower discrimination ratios when compared with the sham group (Figure 5). The current study showed no significant differences in discrimination ratios between group received Aβ. There was significantly lower discrimination ratio in Aβ+s25 group as compared to sham+s25 group (*P* < 0.001) (Figure 5).

In the current study, intra-hippocampal Aβ-injected rats showed memory impairment in novel location test but not in novel object preference test. These observations are in consonance with a former study that showed a lesion of dorsal hippocampus was unable to change the activity of rats in novel object preference test, but was able to impair novel location recognition in same rats^26^ suggesting that the dorsal part of the hippocampus has important role in spatial memory. In addition, Mumby et al observed that rats with hippocampal lesions displayed a novelty preference on object trials but did not discriminate between the objects on place trials or context trials.^32^ These studies indicated that the hippocampal damage impaired memory for contextual or spatial aspects of an experience, whereas memory for objects remained intact. On the contrary, several studies have shown that restricted hippocampal lesions can impair object recognition memory performance.^33,34^

The findings of the present study indicated that spironolactone at all studied doses was unable to improve memory impairment induced by Aβ in rats. No other study has evaluated the effect of spironolactone on Aβ-induced memory impairment to compare our results with. However, there are some studies that showed spironolactone reduced memory. Thus, a study has shown that pre-training spironolactone treatment (50 mg/kg subcutaneous injection) reduced contextual and tone-cue memories in mild foot-shock intensity and fear conditioning tests.^35^ Furthermore, it has been demonstrated that spironolactone (400 mg/d for 3 days before memory test) impaired free remembrance of specific emotional material in young healthy men.^36^ It has been suggested that the blockade of mineralocorticoid receptors (MRs) by spironolactone as a probable cause of memory reduction.^37^ The involvement of MRs in memory has been shown in several studies. Thus, mice with MRs over-expression in forebrain showed significantly decreased neuronal loss after transient cerebral global ischemia improved spatial memory retention in the Morris water maze test and enhanced behavioral response to novelty in the novel recognition test.^37,38^ In addition, Ferguson and Sapolsky showed that rats with over-expression of MRs in the hippocampus displayed an enhancement in memory consolidation in novel recognition tests.^38^ Conversely, there are studies that showed spironolactone can improve memory. Therefore, spironolactone at doses of 50 and 100 mg/kg improved memory impairment following naloxone precipitated withdrawal and reversed memory performance to normal values in spontaneous mice using novel object recognition test.^39^ Moreover, a human study showed that chronic treatment with a low dose of spironolactone (50 mg/d for 6 weeks) improved paired-associated learning in obese individuals suggesting that MRs contributed to hippocampal memory modulation in humans.^16^ In the current study, spironolactone at dose of 25 mg/kg in normal rats did not have any positive or negative effects on memory. Taken together, it seems that spironolactone’s effect on memory is influenced by the cause of memory loss, and type of memory or subjects being studied.

### Iba1 protein level

**Figure 6:**
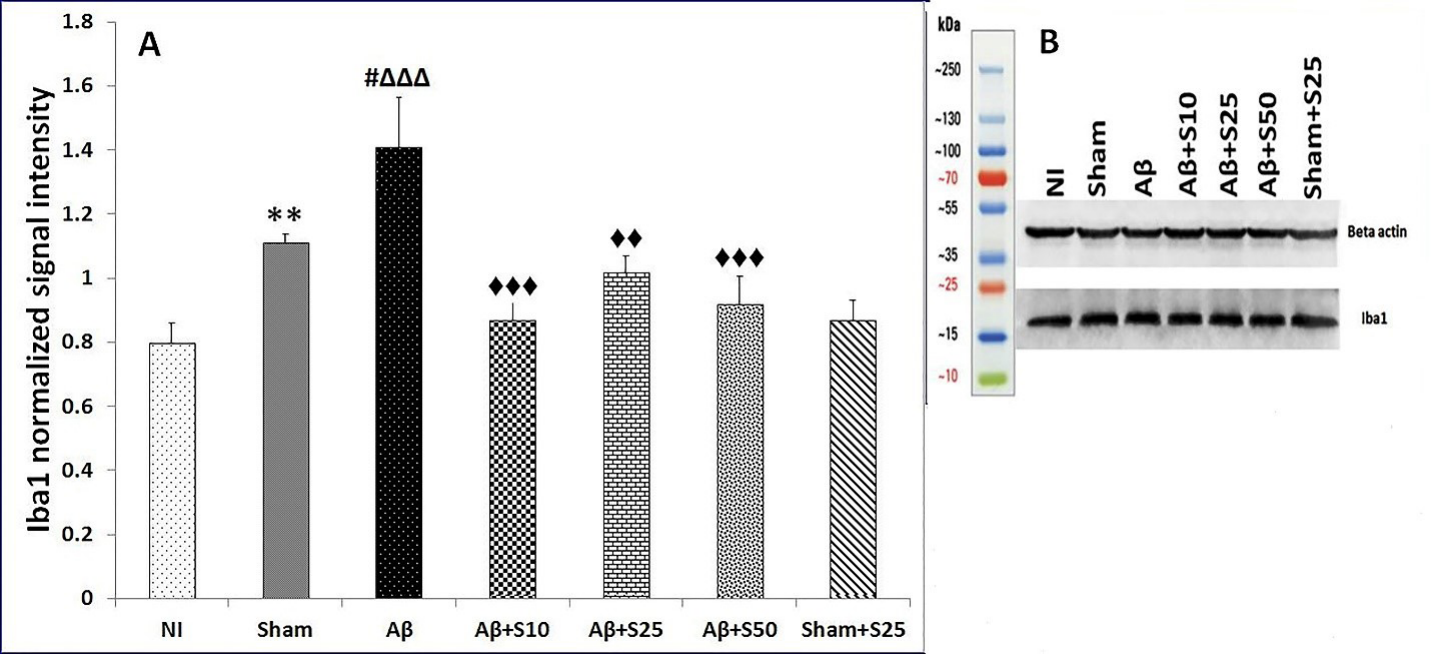
(A) Bar graphs represent the mean + SEM of Iba1 protein levels for no intervention (NI), PBS plus vehicle (sham), Aβ plus vehicle (Aβ), Aβ plus spironolactone 10 mg/kg (Aβ+s10), Aβ plus spironolactone 25 mg/kg (Aβ+s25), Aβ plus spironolactone 50 mg/kg (Aβ+s50) and PBS plus spironolactone 25 mg/kg (sham+s25) (n = 6/group). The level of Iba1 protein was significantly higher in the sham group as compared to the NI group (38.75%, *P* < 0.01). Aβ group had significantly higher Iba1 protein levels as compared to sham (27%, *P* < 0.05) and NI groups (76.2%, *P* < 0.001). Aβ+s10 (38.3%, *P* < 0.001), Aβ+s25 (27.6%, *P* < 0.01), and Aβ+s50 (34.7%, *P* < 0.001) groups had significantly lower Iba1 protein levels in comparison to Aβ group. Moreover, there were no significant differences in Iba1 protein levels between groups receiving different doses of spironolactone. No significant differences in Iba1 protein levels were found between sham and sham+s25 groups (*P* = 0.084). ** P < 0.01 significantly different from NI group; #* P* < 0.05 significantly different from sham group; ∆∆∆* P* < 0.001 significantly different from NI group; ♦♦ *P* < 0.01, ♦♦♦ *P* < 0.001 significantly different from Aβ group. (B) The picture of one blot that represents differences between Iba1 protein levels in seven different groups with compare to beta actin.

The level of Iba1 protein was higher in the sham group in comparison to the NI group (38.75%, *P* < 0.01). Aβ group had higher Iba1 protein levels in reference to sham (27%, *P* < 0.05) and NI groups (76.2%, *P* < 0.001). Aβ+s10 (38.3%, *P* < 0.001), Aβ+s25 (27.6%, *P* < 0.01), and Aβ+s50 (%34.7, *P* < 0.001) groups had significantly lower Iba1 protein levels in comparison to Aβ group (Figure 6). Moreover, there were no significant changes in Iba1 protein levels between groups receiving different doses of spironolactone. Spironolactone did not affect the Iba 1 protein level in comparison to the sham group (*P* = 0.084). No significantly Iba1 protein level was found in Aβ+s25 group in comparison to sham+s25 group (Figure 6).

In the present study, spironolactone decreased hippocampal Iba1 protein levels suggestive of an inhibitory effect of spironolactone on microglial activation. This is in agreement with previous reports regarding anti-inflammatory effects of spironolactone in the peripheral tissues and the CNS.^13,40^ Thus, Sun et al. have suggested that the beneficial effects of spironolactone on radicular pain may be related to the suppression of microglia and pro-inflammatory cytokines in the spinal cord of animals.^18^ In addition, it has been shown that spironolactone at non-toxic concentrations decreased TNF-α in over-activated microglial cells.^41^ The increased Iba1 protein levels in sham group in this study indicated that even the surgery per se can stimulate microglia, however, intra-hippocampal injection of Aβ could further activate microglia as observed by the higher Iba1 protein levels in Aβ group in comparison to the sham group. This is in agreement with previous in vivo and in vitro studies that demonstrated Aβ activated microglial cells^42,43^ which in turn might lead to neurodegeneration. Spironolactone decreased Iba1 protein levels at all studied doses in rats received Aβ and also in sham rats at dose of 25 mg/kg, although not significant. These observations indicated that spironolactone had an inhibitory effect on microglial activation which was more pronounced when marked microglial activation reached due to Aβ injection. Nevertheless, the effect of spironolactone on Iba1 protein levels was not dose dependent. The reason for this finding is currently unclear, however, it is plausible that the doses used in this study were at the saturation, and not at the linear, portion of the spironolactone dose-response curve. This possibility should be examined in future studies using lower and higher doses of spironolactone. On the other hand, the dose independent effect of spironolactone on Iba1 protein levels might be explained by possible direct and indirect effects of spironolactone on microglial activation and neuroinflammation through various mechanisms of actions. Thus, spironolactone may directly inhibit microglial activation by its antagonist action on MRs^44^ which are expressed on microglial cells.^45^ Alternatively, spironolactone exerts antiandrogenic effects and modulates the autophagy system via phosphoinositide-3-kinase–protein kinase B/Akt (PI3K-PKB/Akt) pathway^46,47^ which might have indirectly contributed to the anti-inflammatory action of spironolactone. Therefore, it is likely that the interaction of spironolactone with various receptors and signaling pathways having different actions or sensitivities and/or affinities for spironolactone may lead to dose independent effects of spironolactone on Iba1 protein levels. Further studies are needed to address this notion.

In the current study, spironolactone decreased the level of Iba1 protein levels in Aβ-injected rats with no improving effect on Aβ-induced memory impairment. To explain this finding, one possibility is that the memory impairment caused by Aβ is not directly related to microglial activation and other pathophysiological basis may underlie Aβ-induced memory impairment. As such, the inhibitory effect of spironolactone on microglial activation was not able to overcome the memory deficit caused by Aβ. Another possibility is that the likely positive effects of spironolactone on memory through inhibition of microglial activation were impeded by the negative effect of spironolactone on memory via its MRs blockade action. Nonetheless, the finding that spironolactone inhibited microglial activation in the brain suggest that spironolactone may have neuroprotective effect. In this regard, Guo et al. showed that the neuroprotective effects of donepezil might be related to the suppression of microglial cells.^48^ Future studies should determine neuroprotective effect of spironolactone and its potential beneficial effects on other neurodegenerative disorders involved microglial activation such as Parkinson’s disease, cerebral ischemia or multiple sclerosis.

## Conclusion

The results of this study indicated that spironolactone had a repressive effect on microglial activation with no memory enhancing effect in an animal model of AD. These findings propose that Aβ-induced memory impairment might not be directly related to microglial activation. The inhibitory effect of spironolactone on microglial activation may suggest spironolactone as a potential neuroprotective candidate to be examined in other neurodegenerative disorders.

## Acknowledgments

This study was part of a Ph.D. thesis by Mohammad Mehdipour in partial fulfillment of the requirement for the degree of Ph.D. in Neuroscience. The project was financially supported by a grant (1396-01-55-15023) from Shiraz University of Medical Sciences. The authors would like to thank Ms. Fatema Pirsalami and Ms. Maryam Mojahed Taghi for their technical help.

## Ethical Issues

The ethics committee of the Shiraz University of Medical Sciences approved the study (Ethical code: IR. SUMS. REC.1396. S552).

## Conflict of interest

The authors declare no conflict of interest.
